# Bacteremia among Febrile Ugandan Children Treated with Antimalarials Despite a Negative Malaria Test

**DOI:** 10.4269/ajtmh.14-0494

**Published:** 2015-08-05

**Authors:** Afizi Kibuuka, Pauline Byakika-Kibwika, Jane Achan, Adoke Yeka, Joan N. Nalyazi, Arthur Mpimbaza, Philip J. Rosenthal, Moses R. Kamya

**Affiliations:** Department of Medicine, Makerere University, Kampala, Uganda; Department of Pediatrics and Child Health, Makerere University, Kampala, Uganda; School of Public Health, Makerere University, Kampala, Uganda; Child Health and Development Centre, Makerere University, Kampala, Uganda; Department of Medicine, University of California, San Francisco, California

## Abstract

Bacteremia may be inappropriately treated as malaria in children admitted with a febrile illness in Africa. We determined the prevalence, clinical features, and spectrum of bacteremia among febrile children younger than 5 years of age admitted with a negative malaria test, but prescribed antimalarials at a referral hospital in Jinja, Uganda. After initial evaluation, a blood sample was drawn from 250 children for a complete blood count and bacterial culture. Of 250 samples cultured, 15 grew organisms presumed to be skin contaminants, and of the remaining 235 samples, 45 (19.1%) had bacteremia. *Staphylococcus aureus* (42%), non-typhoidal *Salmonella* (24%), *Pseudomonas aeruginosa* (11%), and *Streptococcus pneumoniae* (9%) were the most common bacterial isolates. On multivariate analysis, history of weight loss (odds ratio [OR] = 2.75; 95% confidence interval [CI] = 1.27–5.95), presence of pulmonary crackles (OR = 3.63; 95% CI = 1.40–9.45), and leukocytosis (OR = 2.21; 95% CI = 1.09–4.47) were independent predictors of bacteremia. At a referral hospital in Uganda, bacteremia was a remarkably common finding in children with febrile illness who were treated for malaria despite negative malaria test results.

## Introduction

In most malaria-endemic countries in Africa, standard practice has been to treat all unspecified febrile illnesses in young children with antimalarial drugs.^1^ However, there are many other causes of febrile illnesses in children, including serious bacterial infections.^2^ For this and other reasons, the World Health Organization (WHO) changed the malaria policy in 2010 from presumptive treatment to parasitological diagnosis and treatment.^3^ In a meta-analysis of 22 studies from Africa, 8.3% of hospitalized children who underwent blood culture had bacteremia, with *Streptococcus pneumoniae* as the commonest isolate, constituting 23.3% of the pathogens.^4^ A study in Kenya found a prevalence of bacteremia in hospitalized children (excluding those with non-medical admissions) of 5.9%, with a minimum annual incidence of community-acquired bacterial infection estimated at 1,080 cases per 100,000 children under 2 years of age.^5^ Another study from Kenya found that 13.2% of children aged 3 months to 12 years with negative tests for malaria had bacteremia, mainly because of non-typhoidal *Salmonella* and *Staphylococcus aureus*.^6^

In resource-limited settings, differentiating between malaria and bacteremia poses a clinical challenge, especially in peripheral centers, leading to the widespread practice of clinical diagnosis without laboratory confirmation.^2^ In Ethiopia, non-malarial febrile illnesses were misdiagnosed as malaria and inappropriately treated with antimalarials due to unavailability of diagnostic facilities and low awareness about non-malarial febrile illnesses.^7^ Many children in Uganda (48%) and Zambia (35%) attending outpatient facilities received antimalarial drugs despite a negative malaria test.^8^,^9^ Wrong diagnosis and treatment poses a number of negative potential consequences, including worsening of the underlying illness, wastage of resources, selection of antimalarial drug resistance, and death.^10^ In Uganda, children admitted to hospital who did not have microscopy performed or had a negative blood smear had a higher risk of death (7.5%) than those with a positive blood smear (3.2%).^11^ In Tanzania, among individuals admitted with a diagnosis of severe malaria, the fatality rate was higher for those with a negative blood smear (12.1%) than those with a positive smear (6.9%).^12^ To better characterize the contribution of bacteremia to acute febrile illnesses in Ugandan children, we conducted a cross-sectional study to determine the prevalence, clinical features, and etiologies of bacteremia among febrile children younger than 5 years of age who were admitted to a referral hospital and treated with antimalarials despite negative malaria tests.

## Materials and methods

### Study area and population.

The study was conducted in the emergency unit of Jinja Children's Hospital, an affiliate of Jinja Regional Referral Hospital, the largest hospital in eastern Uganda. Vaccination against *Haemophilus influenzae* and hepatitis B was introduced at the facility in 2002. During the study period, pneumococcal vaccine was not available. The hospital admits about 1,200 children per month and its catchment area has low to moderate malaria transmission, with an entomological inoculation rate estimated at six infective bites per person-year.^13^ The study was approved by the Makerere University School of Medicine Research and Ethics Committee and the Uganda National Council for Science and Technology.

### Patient recruitment and enrollment.

A study physician based in the emergency unit reviewed medical report forms for all admitted children to assess study eligibility prior to treatment administration. Study eligibility criteria included 1) a Giemsa-stained thick blood smear negative for malaria parasites, 2) antimalarial prescription by an attending physician, 3) age 6 to < 60 months, 4) documented fever (axillary temperature > 37.5°C) or history of fever in the previous 24 hours, 5) no history of antibacterial use within the last 5 days, with the exception of cotrimoxazole prophylaxis for human immunodeficiency virus (HIV)-infected children, and 6) provision of informed consent by a parent or guardian.

### Patient evaluation.

Detailed medical history and physical examination were performed for each child. History of weight loss during the illness as reported by parent/guardian was noted and immunization status was verified on the child health card. Weight was recorded to the nearest 0.1 kg using a scale that was calibrated daily. Height was measured using a vertical stadiometer for children aged 37–59 months and length using a horizontal stadiometer for those aged 6–36 months. Axillary temperature was measured using a digital thermometer. Mid upper arm circumference was measured using a tape measure to determine nutritional status, which was classified as normal (> 12.5 cm), at risk of acute malnutrition (11.5–12.5 cm), and severe wasting (< 11.5 cm). Nutritional status was further classified using weight for height Z-scores as normal (within two standard deviations [SD] of the average), moderate wasting (2–3 SD below average), and severe wasting (> 3 SD below average).^14^

### Laboratory procedures.

To obtain samples, venipuncture sites were disinfected before phlebotomy with 70% alcohol followed by chlorhexidine gluconate, and ∼4 mL of blood was drawn from the cubital vein of each child prior to receiving any medication. Three milliliters of blood was inoculated into a labeled BACTEC™ Peds Plus vial (containing enriched soybean–casein broth with carbon dioxide) whose septum had been swabbed with 70% alcohol, and 1 mL was used for a complete blood count using a Becton Dickinson FACSCalibur Flow Cytometry System (Belgium). Thick smears obtained from finger pricks were stained using 10% Giemsa for 10 minutes and evaluated for the presence of asexual malaria parasites by expert microscopists. All negative smears were read by a second microscopist, and disparities were settled by a third microscopist.

Inoculated culture vials were stored at 25°C and transported to the Makerere University Medical Microbiology Laboratory in Kampala (accredited by the College of American Pathologists). Inoculated vials were placed in the BD BACTEC™ 9120 device for 7 days, with periodic readings to detect production of carbon dioxide. All samples were placed in the BACTEC device within 12 hours of sample collection. Positive cultures by the BACTEC device were Gram stained and subcultured on either chocolate or blood agar to identify bacterial species. In cases of positive BACTEC with negative Gram stain, samples were subcultured on chocolate agar. Positive blood culture results were reported to the treating physician within 7 days from the time of sample collection. All children were empirically treated with antibacterials after drawing blood for culture. Blood culture samples that grew known skin contaminants were considered contaminants, and their results were not reported to the treating physician.

### Statistical methods.

Based on a previous study conducted in Tanzania, we assumed the prevalence of bacteremia in children with febrile illness and a negative malaria test receiving antimalarial drugs would be 17%.^2^ A sample size of 235 was required to detect this prevalence with a precision of 95% and two-sided significance of 0.05. Data were double entered, and verified using Microsoft Access and analyzed using STATA version 10.0 (STATA Corporation, College Station, TX). Data were summarized into frequencies and percentages, and associations between explanatory variables and bacteremia were determined using the ORs for categorical variables. We used logistic regression analysis to determine independent predictors of bacteremia. The backward selection approach *P* value set at < 0.05 was used to select variables to include in the final regression model.

## Results

### Study population.

Between May and August, 2012, 282 children were screened, of whom 250 were enrolled, and 32 (11.3%) excluded. Among the excluded, 25 had received antibacterials within 5 days of admission and 7 did not consent. Fifteen of the 250 (6%) children had presumed sample contamination, of whom 9 (3.6%) grew coagulase negative *Staphylococcus,* and 6 (2.4%) *Micrococcus.*

Of the 235 evaluable children, 130 (55%) were males, and the median age was 15.5 (interquartile range [IQR] = 10–24) months. One hundred forty two (60%) had received a complete dose of antimalarial drugs within 1 week prior to the hospital visit. All children had up-to-date immunization status. Five children were HIV infected and receiving antiretroviral therapy and daily cotrimoxazole as reported by the parents/care taker. All these HIV-infected children had negative blood cultures.

**Figure 1.**
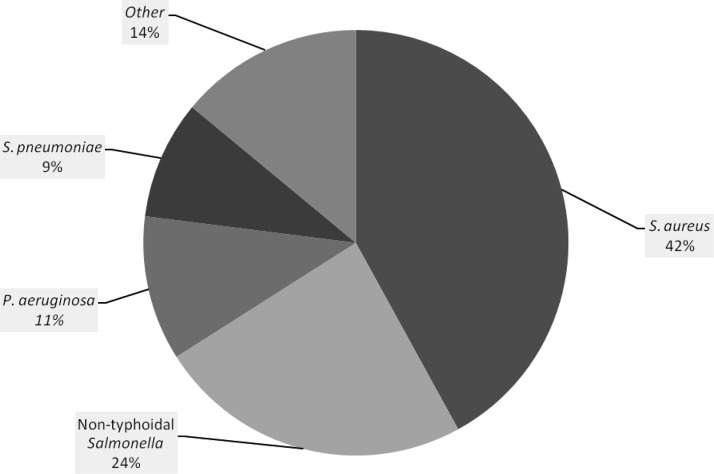
Proportions of bacterial pathogens identified. The proportion of bacteria identified from blood cultures is shown. Other species, each identified in one patient, were *Haemophilus influenzae, Salmonella typhi, Streptococcus viridians, Acinobacter, Citrobacter,* and *Enterococcus.*

Bacteremia was identified in 45 (19.1%) children. The commonest isolate was *Staphylococcus aureus* in 19 (42%) followed by non-typhoidal *Salmonella* in 11 (24%) (Figure 1

). Bacteremia occurred in 23 of 130 boys, and 22 of 105 girls (17.7% versus 21%, *P* = 0.528). Deep breathing, pulmonary crackles, vomiting, and diarrhea were common among children with bacteremia (Table 1). There was no significant difference in the prevalence of anemia between children with or without bacteremia. Neutrophilia and lymphocytosis were more common among children with bacteremia, but differences did not reach statistical significance. Children who presented with a history of weight loss during the illness (OR = 2.58; 95% confidence interval [CI] = 1.31–5.07) or fever duration of 2 or more weeks (OR = 2.35; 95% CI = 1.10–5.02) were more likely to present with bacteremia.

On multivariate analysis, children with a history of weight loss during the illness (OR = 2.75; 95% CI = 1.27–5.95), presence of pulmonary crackles (OR = 3.63; 95% CI = 1.40–9.45), and white blood cell (WBC) count greater than 15,000 cells/μL (OR = 2.21; 95% CI = 1.09–4.47) were more likely to have bacteremia (Table 1).

Of the 217 participants with a known treatment outcome, 212 (97.7%) were alive at discharge while 5 (2.3%) died. All those who died had negative blood cultures.

## Discussion

We evaluated the prevalence, clinical features, and etiology of bacteremia in febrile children admitted to a Ugandan referral hospital with negative malaria smears. We studied children below 60 months of age because young children are known to have high risk of bacteremia.^15^ The prevalence of bacteremia among children with fever and negative malaria blood smear was high.

Our data are consistent with results from previous studies conducted among children with negative malaria smears in Africa. The prevalence of bacteremia was comparable to the prevalence reported in similar settings in Kenya.^6^ It was higher than the prevalence of 8.2% calculated in a meta-analysis on community-acquired blood stream infections in children.^4^

History of weight loss during the illness, presence of pulmonary crackles, and total WBC count > 15,000 cells/μL were independent predictors of bacteremia. Previous studies demonstrated that wasting, respiratory distress, and raised WBC count are common predictors of bacteremia.^16^ In our study, wasting was not found to be a significant predictor, possibly due to the low prevalence of wasting in our study children, likely due to relatively low suspicion for malaria among wasted children, and admission of children with obvious wasting to a nutritional unit, and not the emergency ward, which was the source of subjects for this study. As in other studies conducted in similar settings, *Staphylococcus aureus* and non-typhoidal *Salmonella* were leading causes of bacteremia. A positive association has previously been described between *Staphylococcus aureus* bacteremia and malnutrition,^17^ in contrast to our findings. Non-typhoidal *Salmonella* infection is a common cause of febrile illness among children in malaria-endemic areas of Africa.^4^,^18^ This is likely due, in part, to hemolytic anemia, increased iron availability, accumulation of hemozoin pigment in monocytes and macrophages, and loss of gut integrity^17^ during malaria infection, which together increase susceptibility to infection and impair the cellular immune response. Some studies have demonstrated association of non-typhoidal *Salmonella* bacteremia with low levels of anti-*Salmonella* immunoglobulin G (IgG), mostly in children younger than 2 years of age, suggesting increased susceptibility to infection in young children.^19^ Malnutrition and HIV infection are other reported risk factors for non-typhoidal *Salmonella* infection.^20^ In our study, we did not ascertain HIV serostatus and all children were smear negative for malaria, so we could not assess these potential associations.

*Pseudomonas aeruginosa* is a common cause of hospital acquired bacteremia, which was associated with a high case fatality among hospitalized children in one study from Kenya.^21^ However, we studied children on the day of admission, and so infections were not hospital acquired. We found very low prevalence of *H. influenzae* bacteremia, possibly because our study children had up-to-date immunization. A study in Malawi that evaluated the impact of *H. influenzae* vaccine found a marked decrease in the prevalence of invasive *H. influenzae* infection after vaccine introduction.^22^

Mortality in children with fever and negative blood smears was lower in this study than that reported in some other studies from sub-Saharan Africa,^11^,^12^ possibly because all our study participants received antibacterials empirically. In resource-limited settings, presumptive use of antibacterials in febrile children with negative malaria test results is extensively practiced. Although this may be lifesaving in settings where facilities for blood culture are not available, it may lead to inappropriate use of antibacterials, resulting in selection of drug resistance. Furthermore, indiscriminate prescription of antibacterials limits opportunities for establishing the cause of non-malarial fever and may waste scarce resources.^23^

Our study had some limitations. First, we did not perform Gram stains on samples prior to their placement in the BACTEC instrument; thus, we may have missed cultures that were positive, but had limited growth after the culture was set up. Second, we did not perform blood volume quality control, so we cannot rule out the possibility that some cultures were inoculated with less than 3 mL of blood. Third, we did not ascertain the HIV serostatus of our study children and this limited our ability to demonstrate the effect of HIV infection on bacteremia. Fourth, we relied on microscopy for malaria diagnoses; more sensitive molecular tests may have identified additional parasitemic children, although it is difficult to know whether those with low-level parasitemia and fever are ill because of malaria. Lastly, we relied on reports from the parent/care taker for history of weight loss during illness, which is subjective.

In summary, the prevalence of bacteremia in febrile hospitalized Ugandan children with negative malaria smears was remarkably high. Therefore, this calls for further investigations among febrile children with negative malaria test results.

## Figures and Tables

**Table 1:** Comparison of characteristics between children with and without bacteremia

Variable	Number with bacteremia	Unadjusted		Adjusted	
		OR (95% CI)	*P* value	OR (95% CI)	*P* value
Gender (female)	22	1.23 (0.63–2.36)	0.528	–	–
Age (in months)					
≤ 12	11	Reference	–	–	–
> 12 to < 24	24	1.59 (0.73–3.49)	0.246	–	–
≥ 24	10	1.31 (0.51–3.36)	0.573	–	–
Fever duration > 2 weeks	13	2.35 (1.10–5.02)	0.027	2.05 (0.90–4.68)	0.089
Weight loss during illness	20	2.58 (1.31–5.07)	0.006	2.75 (1.27–5.95)	0.010
Diarrhea	20	1.44 (0.74–2.79)	0.280	–	–
Vomiting	20	1.29 (0.67–2.50)	0.452	–	–
Temperature (≥ 37.5°C)	39	0.48 (0.19–1.21)	0.120	–	–
Deep breathing	14	1.84 (0.89–3.81)	0.099	–	–
Crackles	10	3.07 (1.29–7.33)	0.011	3.63 (1.40–9.45)	0.008
MUAC (< 11.5 cm)	6	1.57 (0.58–4.23)	0.376	–	–
Weight for height					
Normal	38	Reference	–	–	–
Moderate wasting	5	1.02 (0.36–2.90)	0.971	–	–
Severe wasting	2	0.54 (0.12–2.48)	0.431	–	–
WBC > 15,000 cells/μL	23	2.11 (1.09–4.07)	0.026	2.21 (1.09–4.47)	0.027
Hemoglobin level					
Normal (> 11 g/dL)	4	Reference	–	–	–
Mild (9–10.9 g/dL)	16	1.55 (0.48–5.00)	0.463	–	–
Moderate (5.1–8.9 g/dL)	25	2.48 (0.80–7.72)	0.116	–	–
Neutrophilia > 7,700 cells/μL	18	1.70 (0.86–3.30)	0.132	–	–
Leukocytosis (> 4,400 cells/μL)	30	1.52 (0.77–3.01)	0.231	–	–

CI = confidence interval; MUAC = mid upper arm circumference; OR = odds ratio; WBC = total white blood cell count.
